# Giant Cell Arteritis and the Use of Ultrasound in Its Diagnosis

**DOI:** 10.7759/cureus.77283

**Published:** 2025-01-11

**Authors:** Hridya Harimohan, Rohini Bilagi, Quynh Huynh

**Affiliations:** 1 Department of Internal Medicine, Kern Medical, Bakersfield, USA; 2 Department of Internal Medicine, University of California Los Angeles David Geffen School of Medicine, Los Angeles, USA; 3 Department of Rheumatology, Kern Medical, Bakersfield, USA

**Keywords:** colored flow doppler ultrasound, giant cell arteritis (gca), halo, large vessel vasculitis, large vessel vasculitis

## Abstract

Giant cell arteritis is a form of vasculitis causing inflammation of large and medium-sized arteries, including the aorta and its major branches and the temporal artery. Temporal artery biopsy can confirm the diagnosis and should be performed as soon as possible after referral but should not interfere with the initiation of glucocorticoid therapy. A biopsy is typically performed within one week of starting glucocorticoids. However, false negatives may occur due to the segmental nature of giant cell arteritis, inadequate vessel length, or inadequate interpretation. A 60-year-old male with a past medical history of type II diabetes mellitus presented with a headache for the past three days. He did not report any worsening of vision. Physical examination was significant for prominent bilateral temporal arteries and bilateral shoulder, arm, and hip tenderness. Carotid artery duplex ultrasound showed less than 50% stenoses of the internal carotid arteries bilaterally. The patient reported improvement of symptoms, including headache and shoulder and hip pain following steroids. The patient was also continued on prednisone 20 mg and started on tocilizumab 162 mg once weekly. This case report highlights the significance of a duplex ultrasound of carotid arteries as an effective alternative to temporal artery biopsy. Duplex ultrasound is a more sensitive, non-invasive, and cost-effective test and could be considered as an aid in diagnosis.

## Introduction

Giant cell arteritis is a chronic systemic vasculitis affecting large and medium-sized arteries, including the aorta and its major branches and the temporal artery, and is the most common form of systemic vasculitis affecting mostly women more than 50 years old [1]. It is more common among people of Northern European descent. Temporal arteritis is a medical emergency due to the risk of sudden blindness [2]. Forty percent to 60% of patients with giant cell arteritis have polymyalgia rheumatica symptoms while 10% of patients with polymyalgia rheumatica will develop giant cell arteritis [3]. Temporal artery biopsy can confirm the diagnosis and should be performed as soon as possible after referral but should not interfere with the initiation of glucocorticoid therapy. A biopsy is typically performed within one week of starting glucocorticoids; however, a biopsy may demonstrate positive results for two to six weeks after glucocorticoid therapy is initiated [4].

This case report was presented as a poster in the Clinical Congress of Rheumatology, West, 2024.

## Case presentation

A 60-year-old male with a past medical history of type II diabetes mellitus presented with a headache for the past three days. He presented with a stabbing type of headache in the bilateral frontal, temporal, and posterior infra-occipital regions, worse in the right temporal area. The pain was constant in nature, moderate to severe in intensity, and aggravated while moving the jaw, with minimal improvement to over-the-counter analgesics. He denied any aura prior to the headache, associated nausea, vomiting, photophobia, or phonophobia.

He did not report any visual disturbances. The patient also reported muscle pain and stiffness, particularly in the neck, shoulders, upper arms, and hips, bilaterally, worse at night when he rested. Vitals were stable, with a blood pressure of 110/65 mmHg. His pulse rate was 82 beats per minute, respiratory rate was 16 per minute, and temperature was 36.1 °C. Physical examination was significant for prominent and tender bilateral temporal arteries. Bilateral shoulder, arm, and hip tenderness were also present. Laboratory markers are listed in Table 1.

**Table 1:** Laboratory markers

Laboratory Markers (Unit)	Value	Reference Range
Erythrocyte Sedimentation Rate (mm/hour)	49	Less than 20
C-Reactive Protein (mg/dl)	0.56	Less than 0.9
Anti-Nuclear Antibody Titer	1:80	Less than 1:40
Anti-Nuclear Antibody Pattern	Nuclear dense pattern	NA

A clinical diagnosis of giant cell arteritis associated with polymyalgia rheumatica was made. The patient was started on prednisone 20 mg three times daily. MRI brain without contrast showed no evidence of infarction, bleed, or mass. CT head without contrast was unremarkable without acute infarction, intracerebral hemorrhage, or subdural hematoma. A carotid artery duplex ultrasound was done.

**Figure 1:**
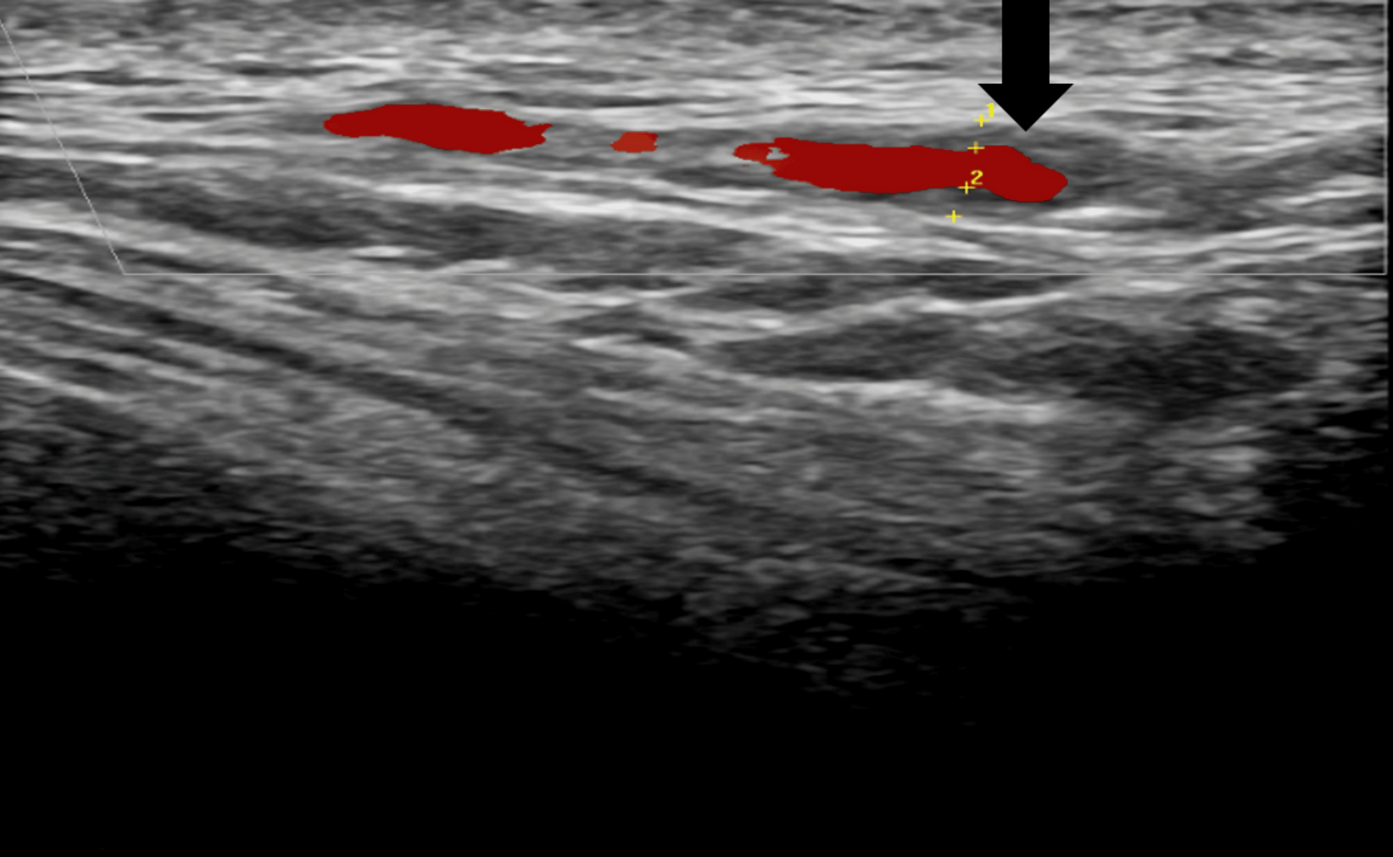
Halo sign seen in the distal temporal artery (marked) in the carotid artery duplex ultrasound

Carotid artery duplex ultrasound showed less than 50% stenoses of the internal carotid arteries bilaterally. A halo sign (Figure 1) was noted along the course of the right temporal artery consistent with giant cell vasculitis. The patient reported improvement of symptoms, including headache and shoulder and hip pain following steroids. The patient was also continued on prednisone with a plan to start on tocilizumab 162 mg once weekly for optimal glucose control because of the past medical history of diabetes mellitus.

## Discussion

Once giant cell arteritis is suspected, it is essential to start the patient on steroids before confirming the diagnosis with a temporal artery biopsy [4,5]. Biopsy when performed within one week of starting glucocorticoids ideally shows positive results. However, false-negative results, ranging from 9% to 61%, may occur due to the segmental nature of giant cell arteritis, inadequate vessel length, or inadequate interpretation [5,6]. Duplex ultrasound is a more sensitive, non-invasive, and cost-effective test and could be considered as an aid in diagnosis. Studies have shown that several European rheumatology centers have equipped themselves with fast-track giant cell arteritis clinics, which consist of same-day ultrasound and initiation of treatment with the relative risk of permanent blindness in the giant cell arteritis patients diagnosed through the fast-track clinic, 88% lower compared to those diagnosed by the conventional route via biopsy with a shorter mean duration of inpatient care by 3 days [7,8].

## Conclusions

This case report highlights the significance of duplex ultrasound of carotid arteries as an effective alternative to temporal artery biopsy. The availability of temporal artery biopsy in a timely manner after the initiation of steroids is questionable, especially in resource-limited settings. Also, it is extremely essential to have a prompt diagnosis of giant cell arteritis, as it would aid in the necessity of long-term steroids or immunosuppressants as clinically important. Hence, because of the risks of false-negative results in the biopsy of temporal arteries, duplex ultrasound is an effective alternative.
